# Cohesin-Dependent Association of Scc2/4 with the Centromere Initiates Pericentromeric Cohesion Establishment

**DOI:** 10.1016/j.cub.2013.02.022

**Published:** 2013-04-08

**Authors:** Josefin Fernius, Olga O. Nerusheva, Stefan Galander, Flavia de Lima Alves, Juri Rappsilber, Adele L. Marston

**Affiliations:** 1 The Wellcome Trust Centre for Cell Biology, School of Biological Sciences, Michael Swann Building, Mayfield Road, Edinburgh EH9 3JR, UK

## Abstract

Cohesin is a conserved ring-shaped multiprotein complex that participates in chromosome segregation, DNA repair, and transcriptional regulation [1, 2]. Cohesin loading onto chromosomes universally requires the Scc2/4 “loader” complex (also called NippedBL/Mau2), mutations in which cause the developmental disorder Cornelia de Lange syndrome in humans [1–9]. Cohesin is most concentrated in the pericentromere, the region surrounding the centromere [10–15]. Enriched pericentromeric cohesin requires the Ctf19 kinetochore subcomplex in budding yeast [16–18]. Here, we uncover the spatial and temporal determinants for Scc2/4 centromere association. We demonstrate that the critical role of the Ctf19 complex is to enable Scc2/4 association with centromeres, through which cohesin loads and spreads onto the adjacent pericentromere. We show that, unexpectedly, Scc2 association with centromeres depends on cohesin itself. The absence of the Scc1/Mcd1/Rad21 cohesin subunit precludes Scc2 association with centromeres from anaphase until late G1. Expression of *SCC1* is both necessary and sufficient for the binding of cohesin to its loader, the association of Scc2 with centromeres, and cohesin loading. We propose that cohesin triggers its own loading by enabling Scc2/4 to connect with chromosomal landmarks, which at centromeres are specified by the Ctf19 complex. Overall, our findings provide a paradigm for the spatial and temporal control of cohesin loading.

## Graphical abstract

**Graphical Abstract:**

**Figure:**
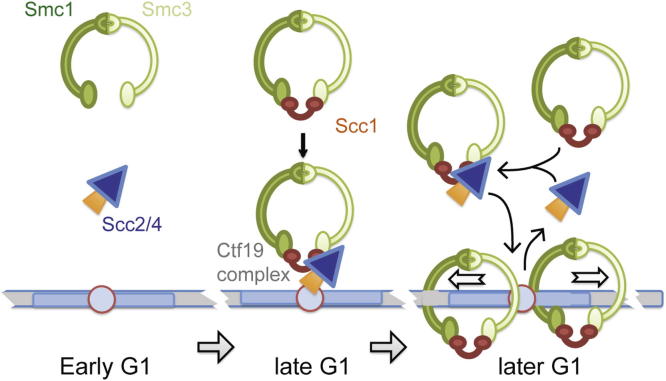

**Highlights:**

► Cohesin is required for the association of its loader, Scc2/4, with centromeres ► The Ctf19 kinetochore subcomplex targets the loader to centromeres ► Centromere-loaded cohesin ensures pericentromeric cohesion establishment ► Cohesin ring formation triggers cohesin loading via Scc2/4

## Results and Discussion

### Scc2 Association with Centromeres Depends on the Ctf19 Complex

Cohesin is highly enriched throughout the pericentromere, yet the Scc2/4 cohesin loader shows strong enrichment only within the core ∼125 bp centromere [10, 19–21] (see Figures S1A and S1B available online). Enrichment of Scc2 at centromeres, but not at a control tRNA site [21, 22], requires Ctf19 complex components [17] (Figures S1C and S1D). Scc2 turns over rapidly near kinetochores [20] and does not stably associate with the Ctf19 complex (Figures S1E, S1F, and Table S3). Even when the Ctf19 complex was purified from cells producing a version of Smc3 (Smc3E1155Q) blocked at an early step in cohesin loading [20, 23], virtually the entire kinetochore, yet only very few peptides of cohesin and its loader, were recovered (Figure S1E and Table S3). These data suggest that the Ctf19 complex promotes cohesin enrichment throughout the pericentromere by enabling the transient association of the Scc2/4 cohesin loader with centromeres.

### Centromere-Tethered Scc4 Is Sufficient to Recruit Scc2 and Scc1

**Figure 1:**
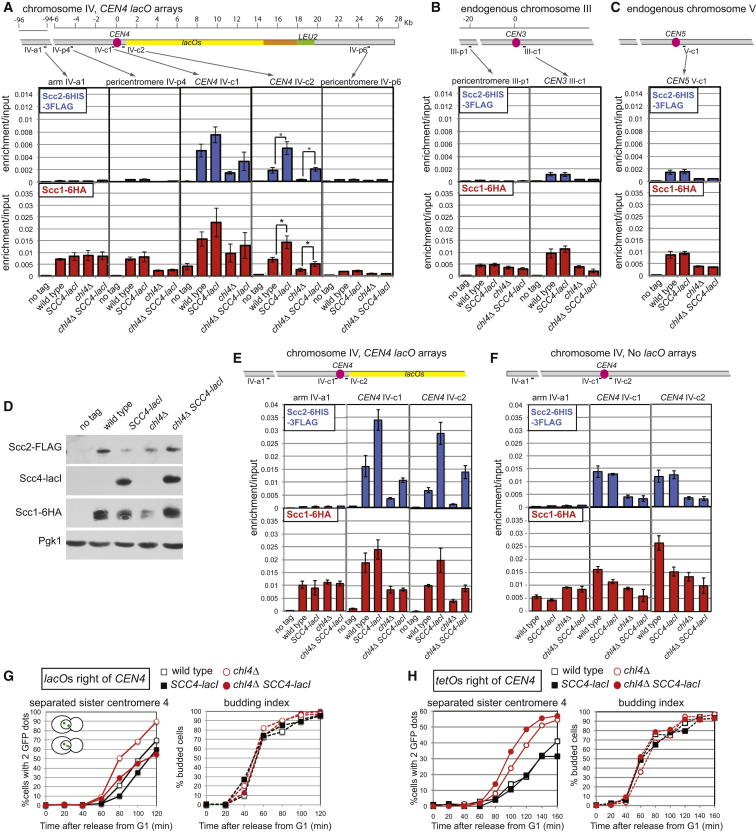
The Ctf19 Complex Promotes Cohesion Establishment by Enabling Association of the Scc2/4 Loader with Centromeres (A–D) Artificial recruitment of Scc2/4 to a centromere leads to a modest increase in Scc1 levels at the tethering site. An approximately 10 kb array of *lacO* repeats was integrated approximately 500 bp to the right of *CEN4* in strains that were otherwise wild-type (AM10285), *pGAL-SCC4-lacI* (AM10287), *chl4Δ* (AM10289), or *chl4Δ pGAL-SCC4-lacI* (AM10291) and carried *SCC2-6HIS-3FLAG* and *SCC1-6HA* as well as a no-tag control (AM8387). Strains were precultured in raffinose-containing medium and then arrested in nocodazole and benomyl for 2.5 hr in medium containing raffinose and galactose before harvesting for Scc1 and Scc2 ChIP. qPCR analysis (A–C) using the indicated primer sets on chromosome IV (A), III (B), or V (C). The mean of four independent experiments is shown with error bars representing standard error (^∗^p < 0.5, two-tailed paired t test). Anti-FLAG, anti-lacI, anti-HA, and anti-Pgk1 (D) immunoblots showing relative levels of Scc2-6HIS-3FLAG, Scc4-LacI, Scc1-6HA, and Pgk1 (loading control) in the samples analyzed from a representative experiment. (E and F) Recruitment of Scc1 and Scc2 by Scc4-LacI is dependent on *lacOs*. Strains with a 10 kb array of *lacO* repeats ∼500 bp from *CEN4* (E) or without *lacOs* (F) and with the indicated genotypes were grown and analyzed as in (A), and Scc1 and Scc2 ChIP is shown for the indicated primer sets. Strains with *lacOs* (E) are as in (A). Strains without *lacOs* (F) all carried *SCC2-6HIS-3FLAG* and *SCC1-6HA* and are otherwise wild-type (AM8413), *pGAL-SCC4-lacI* (AM11066), *chl4Δ* (AM8415), *chl4Δ pGAL-SCC4-lacI* (AM11065). Note that the presence of *lacO* arrays adjacent to *CEN4* reduces cohesin association with IV-c2. Mean of three independent experiments is shown and error bars indicate standard error. (G) Tethering Scc2/4 to centromeres rescues the centromeric cohesion defect of *chl4Δ* cells. Strains with approximately 10 kb *lacO* arrays integrated adjacent to *CEN4*, producing LacI-GFP and carrying *pMET-CDC20*, were arrested in G1 in raffinose-containing medium lacking methionine by treatment with alpha factor before being released into medium containing raffinose, galactose, and methionine to induce a metaphase arrest and induce Scc4-LacI. Samples were taken at the indicated time points after release from alpha factor, and the number of GFP foci per cell and budding was scored. A representative experiment is shown. Strains used were AM10570 (wild-type), AM10571 (*pGAL-SCC4-lacI*), AM10572 (*chl4Δ*), and AM10573 (*chl4Δ pGAL-SCC4-lacI*). (H) Scc4-lacI requires *lacO* arrays at *CEN4* to rescue the cohesion defect of *chl4Δ* cells. To visualize *CEN4*, while preventing Scc4-lacI binding, *tetO* arrays were integrated approximately 2.4 kb right of *CEN4* in *pMET-CDC20* strains producing TetR-GFP. Wild-type (AM4643), *SCC4-lacI* (AM11162), *chl4Δ* (AM4644), and *chl4Δ SCC4-lacI* (AM11163) were treated as described in (G), and a representative experiment is shown. See also Figure S1.

To test this idea, we reconstituted the Scc2/4 cohesin loader at a centromere in the absence of a functional Ctf19 complex. We produced Scc4 fused to bacterial LacI in wild-type and *chl4Δ* strains carrying *lacO* repeats integrated adjacent (∼500 bp) to the centromere on chromosome IV (*CEN4*; Figures 1A and 1D). At a site (IV-c2) directly adjacent to the *CEN4*-proximal *lacOs*, production of Scc4-LacI reproducibly increased the levels of the cohesin loader (Scc2) and restored Scc1 to close to wild-type levels in *chl4Δ* cells (Figure 1A). *CEN3*, *CEN5*, and a chromosomal arm site showed no increased Scc1 or Scc2 enrichment upon Scc4-LacI production, indicating that the Scc4-LacI tether specifically recruited cohesin in its vicinity (Figures 1B and 1C). In the absence of *lacOs*, Scc4-LacI production slightly decreased Scc1 at tested chromosomal sites, including IV-c2, though Scc2 levels remained unchanged (Figures 1E and 1F). Although this suggests that untethered Scc4-LacI might interfere with overall cohesin loading by endogenous Scc4, it confirms that Scc4-LacI only recruits Scc1 and Scc2 when tethered to *lacOs*. Interestingly, centromeric or pericentromeric sites more distant from the *lacOs* on chromosome 4 did not show appreciable increases in either Scc1 or Scc2 levels upon Scc4-lacI production, and tethered Scc4-LacI recruited Scc2 more efficiently than Scc1 (Figure 1A). Therefore, although tethered Scc2/Scc4-LacI can recruit Scc1 in a localized manner in *chl4Δ* cells, it fails to fully recapitulate wild-type pericentromeric cohesin enrichment. This indicates that tethered Scc4-lacI lacks dynamic interactions that enable cohesin translocation. In addition, the Ctf19 complex may affect pericentromeric cohesin recruitment in ways other than targeting Scc2/4 to centromeres.

### Centromere-Tethered Scc4 Rescues the Cohesion Defect of *chl4Δ* Cells

Although *CEN4*-tethered Scc4-LacI led to only a localized increase in Scc1, cohesin recruited to the *lacOs* might rescue the cohesion defect of *chl4Δ* cells at *CEN4*. We produced LacI-GFP in wild-type and *chl4Δ* cells carrying *CEN4-*proximal *lacOs* and tested the effect of Scc4-lacI production on *CEN4-*GFP separation as cells progressed from G1 into metaphase (Figure 1G). Analysis of budding indicated similar cell-cycle timing in all strains, but *chl4Δ* cells separated sister centromeres prematurely and to a greater extent than wild-type cells (Figure 1G). Remarkably, production of Scc4-LacI reduced the frequency of separated foci in *chl4Δ* cells to a level close to that of wild-type. Similarly, Scc4-LacI production improved the cohesion of wild-type *CEN4* (Figure 1G). This was a specific effect of tethering Scc4-lacI nearby, because cohesion at *CEN4* was not improved when Scc4-lacI was produced in strains without *lacOs*, where *CEN4* was visualized with TetR-GFP bound to *tetO* arrays (to which Scc4-lacI cannot bind) (Figure 1H). These results indicate that a failure to recruit Scc2 to centromeres, thereby abrogating cohesin loading, causes defective centromeric cohesion in *chl4Δ* cells.

### De Novo Cohesin Loading at the Centromere

**Figure 2:**
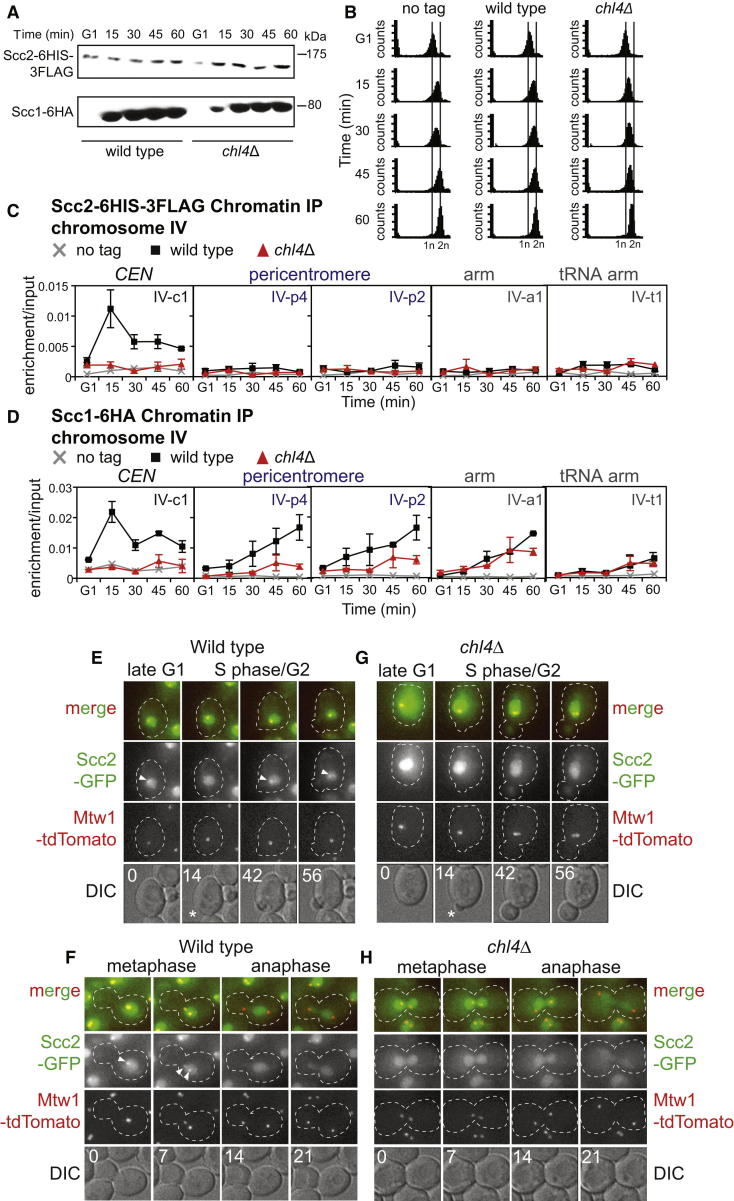
Scc2 Associates with Centromeres from Late G1 until Anaphase (A–D) Sites of association of Scc1 and Scc2 as cells progress into the cell cycle and their dependence on Chl4. Strains AM1176 (no tag), AM8414 (*SCC2-6HIS-3FLAG*), and AM8415 (*SCC2-6HIS-3FLAG, chl4Δ*) were arrested in G1 in alpha factor at room temperature, then released into medium containing benomyl and nocodazole at 18°C. Samples were extracted prerelease (G1) and at 15, 30, 45, and 60 min following release for analysis of Scc1-6HA and Scc2-6His-3FLAG levels by western blotting (A), analysis of DNA content by FACS (B), and association of Scc1-6HA and Scc2-6His-3FLAG with the indicated sites by ChIP-qPCR (C and D). The mean of three independent experiments is shown, with error bars representing standard error. (E–H) Live-cell imaging showing Scc2 localization during the cell cycle and its dependence on Chl4. Still images from movies of wild-type (E and F; AM10713) and *chl4Δ* (G and H; AM10714) cells carrying Scc2-GFP and Mtw1-tdTomato. The numbers on the DIC images represent minutes relative to the first image. Arrowheads and asterisks indicate the position of the Scc2-GFP associated with kinetochores and the newly forming bud, respectively. See also Figure S2 and Movies S1, S2, S3, and S4.

Because neither cohesin (Scc1) nor its loader (Scc2) are localized to chromosomes in G1-arrested cells [19, 24], we asked whether the Ctf19 complex is required for de novo cohesin loading upon cell-cycle entry. Scc2 levels are unchanged throughout the cell cycle, whereas Scc1 appears only upon cell-cycle entry [19, 24] (Figures 2A and 2B). In G1-arrested cells, as expected, neither Scc1 nor Scc2, associated with five chromosomal sites tested (Figures 2C and 2D). However, upon cell-cycle entry, both Scc1 and Scc2 were rapidly recruited to chromosomes, though their pattern was distinct. Scc2 was most enriched at the centromere, was not detected in the pericentromere or at a “high-cohesin” chromosomal arm site, and was present at only very low levels at a tRNA site (Figure 2C). In contrast, Scc1 associated with the centromere, pericentromere, and an arm site, and low levels were also observed at the tRNA site (Figure 2D). Both Scc1 and Scc2 associated with centromeres immediately upon cell-cycle entry (Figures 2C and 2D). However, association of Scc1 with sites distant from its loader (i.e., pericentromeres, chromosomal arm) occurred later. This is consistent with the idea that cohesin loads onto defined chromosomal sites, such as centromeres, and then subsequently translocates away from these sites into the adjacent part of the chromosome [10, 20]. Taken together with the high dynamicity of Scc2 at centromeres [20], it seems likely that Scc2/4 dissociates from the chromosome following the loading reaction.

Deletion of *CHL4* abolished the recruitment of both Scc1 and Scc2 to centromeric, but not chromosomal arm, sites (Figures 2C and 2D). Consistent with the idea that pericentromeric cohesin is derived mainly from that loaded at centromeres, cohesin accumulation within the pericentromere was also greatly reduced in the *chl4Δ* mutant. Interestingly, however, low levels of cohesin appeared in the pericentromere after a delay (Figure 2D). Although this could be explained by weak loading activity in the pericentromere, we favor the idea that it travels from loading sites outside the pericentromere [10].

### Scc2 Association with Kinetochores Is Dependent on Chl4

We confirmed the cell-cycle regulation of Scc2 association with centromeres and its dependence on the Ctf19 complex by time-lapse microscopy of live cells carrying Scc2-GFP and the kinetochore marker Mtw1-tdTomato (Figures 2E–2H; Movies S1, S2, S3, and S4). In wild-type cells, Scc2-GFP showed enrichment at kinetochores from late G1 until metaphase, though nuclear localization was observed at all cell-cycle stages (Figures 2E and 2F). Interestingly, the kinetochore-associated Scc2-GFP signal disappeared in anaphase and was not observed in early G1 cells (Figures 2E and 2F). In *chl4Δ* cells, Scc2-GFP was localized in the nucleus, but kinetochore-associated foci were absent at all stages of the cell cycle (Figures 2G and 2H). We conclude that Scc2 association with centromeres is subject to temporal control in addition to its spatial regulation by the Ctf19 complex.

### *SCC1* Expression Is Required for Scc2 Association with Centromeres

What controls the timing of Scc2 association with centromeres? We ruled out DNA replication as a possible cause, given that cohesin can associate with unreplicated chromosomes [25] (Figure S2). Another possibility is that the cohesin subunit, Scc1, which is absent in G1 and cleaved in anaphase [24, 26, 27] (Figure 2A) is required for Scc2 association with centromeres. Previous experiments using temperature-sensitive versions of cohesin subunits are difficult to interpret, given that we found that centromeric Scc2 levels are affected by temperature even in wild-type cells [19] (Figure S3).

**Figure 3:**
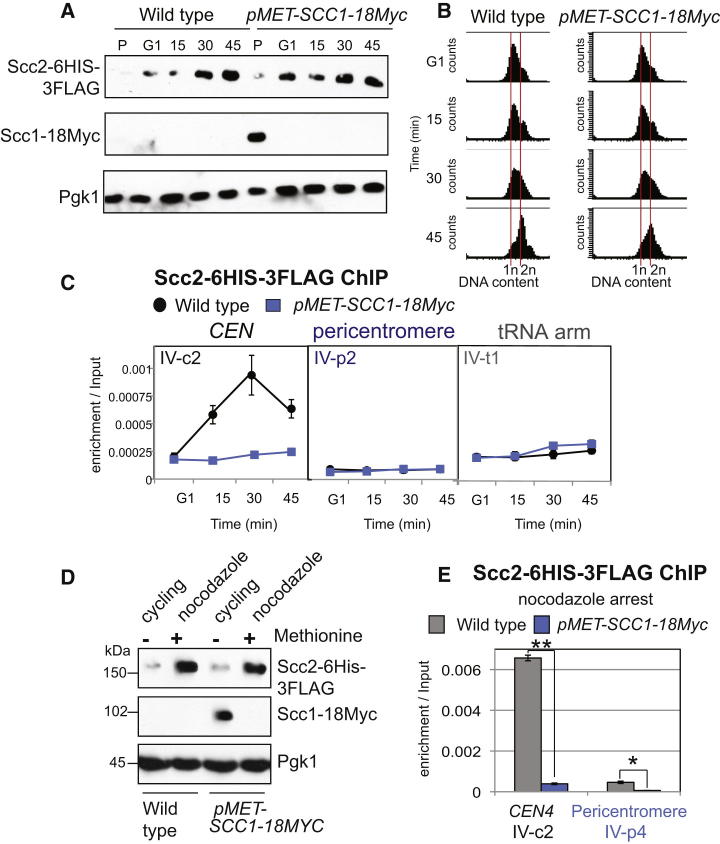
Scc1 Is Required for Scc2 Association with Centromeres (A–C) Analysis of Scc2 association with centromeres upon cell-cycle entry in the absence of Scc1. Strains AM6006 (*SCC2-6HIS-3FLAG*) and AM9519 (*SCC2-6HIS-3FLAG pMET-SCC1-18MYC*) were arrested in G1 with alpha factor in rich medium containing methionine to deplete Scc1 for 3 hr and then released into methionine-containing medium. Samples were extracted at 0 (G1), 15, 30, and 45 min after release for analysis of protein levels by anti-FLAG, anti-Myc, and anti-Pgk1 immunoblot (A), analysis of DNA content by FACS (B), and protein localization at the indicated sites by ChIP-qPCR (C). (D and E) Analysis of Scc2 association with centromeres in nocodazole-arrested cells after Scc1 depletion. Strains as in (A)–(C) were arrested in alpha factor in the presence of methionine for 3 hr before being released into methionine- and nocodazole-containing medium for 2 hr. Immunoblot (D) showing protein levels at the time of harvesting (nocodazole) and preincubation with alpha factor (cycling). Analysis of ChIP samples by qPCR (E) at the indicated sites. The mean of three independent experiments is shown. Error bars represent standard error (^∗∗^p < 0.01; ^∗^p < 0.05, two-tailed paired t test). See also Figure S3.

Instead, we analyzed Scc2 association with chromosomes after Scc1 depletion. Endogenous *SCC1* was placed under the methionine-repressible promoter and Scc2 localization was analyzed as cells entered the cell cycle in the presence of methionine to prevent *SCC1* expression. We confirmed that Scc2 was present at all time points, that Scc1 was successfully depleted in *pMET-SCC1-18MYC* cells, and that both strains entered the cell cycle with similar timing (Figures 3A and 3B). Although Scc2 was loaded onto the centromere in wild-type cells, its levels did not rise above background in Scc1-depleted cells (Figure 3C). Similar results were obtained in metaphase-arrested cells (Figures 3D and 3E). We conclude that *SCC1* expression is required for the association of Scc2 with centromeres.

### Scc1 Production in G1 Is Sufficient to Trigger Scc2 Docking and Cohesin Loading

**Figure 4:**
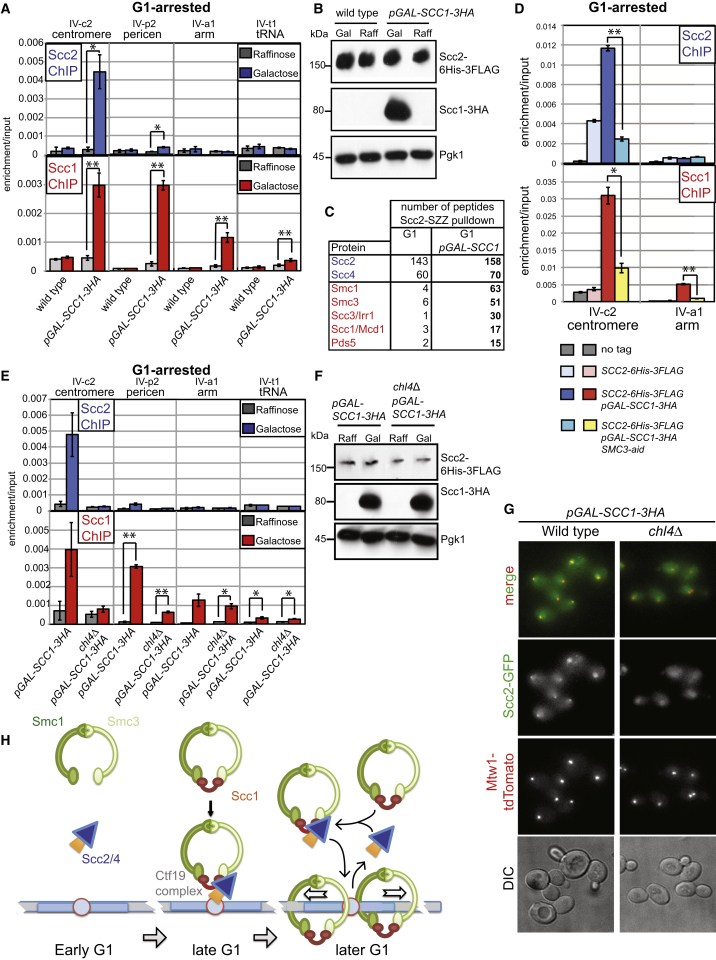
*SCC1* Expression Is Sufficient for Scc2 to Associate with Centromeres and for Scc1 Loading in G1 (A and B) Strains AM9335 (*SCC2-6HIS-3FLAG*) and AM9334 (*SCC2-6HIS-3FLAG pGAL-SCC1-3HA*) were arrested in G1 in raffinose-containing medium with alpha factor for 3 hr. Samples were extracted for anti-FLAG, anti-HA, and anti-Pgk1 immunoblotting (B) and ChIP-qPCR (A; raffinose), then 2% galactose was added to the remainder, while maintaining the G1 arrest. After 45 min the remainder of the culture was harvested for immunoblotting (B) and ChIP-qPCR (A; galactose). (C) Mass spectrometry analysis of proteins copurifying with Scc2 after immunoprecipitation from G1-arrested cells with and without *SCC1* expression. The number of identified peptides for the indicated proteins is shown. (D) Smc3 is required for Scc2 and Scc1 to associate with centromeres. Strains AM1176 (no tag), AM6006 (*SCC2-6HIS-3FLAG*), AM9334 (*SCC2-6HIS-3FLAG pGAL-SCC1*), and AM11092 (*SCC2-6HIS-3FLAG pGAL-SCC1 SMC3-aid*) were arrested in G1 in raffinose-containing medium with alpha factor for 3 hr before addition of 0.5 mM NAA. After 30 min, while maintaining G1 arrest, 2% galactose was added, and cultures were harvested for Scc2 and Scc1 ChIP after 45 min. (E and F) Ectopic loading of cohesin in G1 is dependent on Chl4. Analysis of Scc1 and Scc2 loading at centromeres in G1-arrested wild-type and *chl4Δ* mutant cells, following ectopic expression of Scc1. Strains AM9334 (*SCC2-6HIS-3FLAG pGAL-SCC1-3HA*) and AM9613 (*SCC2-6HIS-3FLAG, pGAL-SCC1-3HA chl4Δ*) were treated as in (A) and (B), and qPCR analysis of ChIP samples (E) and immunoblot (F) are shown. (G) Scc1 production enables Scc2 association with kinetochores throughout the cell cycle. Images are shown of live wild-type (AM10715) and *chl4Δ* cells (AM10716) carrying *pGAL-SCC1-3HA*, *SCC2-GFP*, and *MTW1-tdTomato* after culturing in galactose-containing medium for 2 hr. (H) Model for the spatial and temporal control of cohesin loading by Scc1 and the Ctf19 complex. In (A), (D), and (E) the mean of three independent experiments is shown with error bars representing standard error (^∗∗^p < 0.01; ^∗^p < 0.05, two-tailed paired t test). See also Figure S4 and Table S3.

Is Scc1 expression the key event that triggers cohesin loading upon cell-cycle entry? If so, ectopic production of Scc1 in G1-arrested cells would be predicted to trigger Scc2 association with the centromere and cohesin loading. To allow expression of *SCC1* in G1, we integrated an additional copy of *SCC1* carrying a 3HA tag under control of the galactose-inducible promoter (*pGAL-SCC1-3HA*). As expected, in G1-arrested cells grown in raffinose, Scc1-3HA was absent, though Scc2 was produced (Figure 4B) and neither protein localized to chromosomes (Figure 4A). However, addition of galactose to induce *pGAL-SCC1-3HA* during the G1 arrest drove Scc1-3HA production and, despite residual separase activity in early G1 cells, robust full-length Scc1 production (Figures 4B and S4A). Remarkably, Scc1 production led to the association of both Scc2 and Scc1-3HA with the centromere (Figures 4A and 4B). Similar to the loading of endogenous cohesin, ectopic G1-loaded Scc1-3HA was found in the pericentromere (Figure 4A). Furthermore, ectopic G1-loaded Scc1-3HA also associated with a chromosome arm site where endogenous cohesin normally resides (Figure 4A), suggesting that Scc1 production in G1 triggers transient Scc2 association with sites throughout the genome, though we are only able to detect it reliably at the centromere among the sites tested. Taken together with the data shown in Figure 3C, these findings show that *SCC1* expression upon cell-cycle entry is both necessary and sufficient for Scc2 association with centromeres, at least, and cohesin loading throughout the genome. These findings refute the view that the Scc2/4 complex is established on centromeres prior to binding cohesin and rather suggest that Scc2 associates with chromosomes only during the act of cohesin loading.

### Production of Scc1 Promotes the Association of Cohesin with Its Loader

The Smc1 and Smc3 subunits of cohesin associate to produce a V-shaped structure, but association of the Scc1 subunit is required to close the ring [28] and allow its association with chromosomes [20]. We hypothesized that cohesin ring formation upon Scc1 expression in G1 might allow Scc2/4 binding, enabling the entire complex to associate with chromosomes and the cohesin loading reaction to occur. This idea predicts that cohesin subunits associate with the Scc2/4 loader only in the presence of Scc1. To test this possibility, we immunoprecipitated Scc2-6His-3FLAG either from wild-type cells or *pGAL-SCC1*-*3HA* G1-arrested cells in the presence of galactose. Analysis of protein complexes copurifying with Scc2 by mass spectrometry revealed many more peptides corresponding to cohesin subunits (Smc1, Smc3, Scc1, Scc3, Pds5) in Scc1-expressing cells, though cohesin loader (Scc2, Scc4) peptides were recovered with similar numbers from both strains (Figure 4C; Table S3). This indicates that Scc1 enables the entire cohesin ring to associate with its loader. To test whether other cohesin subunits are required for Scc2 association with centromeres, we tagged Smc3 with the auxin-inducible degron (aid) [29]. Treatment of G1-arrested Smc3-aid cells with auxin (NAA), triggered Smc3-aid degradation and greatly reduced the levels of Scc1 and Scc2 recruited to centromeres upon ectopic Scc1 production (Figures 4D and S4B). These results indicate that cohesin ring formation promotes the association of the Scc2/4 cohesin loader with centromeres.

### Ectopic Scc2 Loading at Centromeres Requires Chl4

We asked whether the spatial information provided by the Ctf19 complex is also required for Scc2 to associate with centromeres during ectopic Scc1-induced G1 loading. Although Scc1 production in G1 triggered Scc2 binding to the centromere in wild-type cells, it failed to do so in *chl4Δ* cells (Figures 4E and 4F). However, levels of ectopically produced Scc1 at a chromosomal arm site were similar in wild-type and *chl4Δ* cells, indicating that only the centromeric loading site was affected by *chl4Δ*. Interestingly, low levels of Scc1 were also detected in the pericentromere in *chl4Δ* cells under these conditions (Figure 4E), again supporting the idea that cohesin loaded at sites distant from the centromere can travel into the pericentromere in *chl4Δ* cells.

Live-cell microscopy of wild-type and *chl4Δ* cells ectopically expressing *SCC1* confirmed that Scc2 can associate with kinetochores at all cell-cycle stages in a Chl4-dependent manner (Figure 4G). We conclude that Scc1 production is the limiting factor for cohesin loading during the cell cycle.

### Requirements for Cohesin Loading

We have defined the requirements for cohesin loading onto centromeres by Scc2/4. We show that cohesin loading at centromeres is defined temporally by the availability of the Scc1 subunit of cohesin and spatially by the Ctf19 complex. Scc2/4 is not prebound to centromeres, but rather Scc2/4 and cohesin bind coordinately. It seems likely that cohesin ring formation is similarly required for Scc2/4 association with its other, much weaker, sites of association on chromosome arms. This suggests a model that invokes Scc2/4 as an accessory factor that both connects cohesin with spatial landmarks and enables its release onto the chromosome (Figure 4H). This draws parallels to the loading of sliding clamps during DNA replication where prebinding of the loader opens the clamp and triggers DNA binding, thereby stimulating the ATPase activity of the loader and release of the clamp [30]. Cohesin itself, rather than its loader, hydrolyzes ATP; nevertheless, this is important not for cohesin association with loading sites but rather its translocation away from these sites [20]. Our findings imply the requirement for a docking site for the Scc2/4-cohesin complex to perform this loading reaction. Clearly, the predominant docking site is at centromeres, defined by the Ctf19 complex, though weaker, less-defined sites must exist on chromosome arms [22]. Presumably, new cohesin loading sites must also be set up in response to a need to generate new cohesive domains to drive cohesin-dependent alterations in transcription or repair of DNA lesions [5–7]. The ability to spatially and temporally regulate cohesin loading might be especially critical in these contexts where dynamic chromatin interactions are required.

## Supplemental Information

Document S1. Figures S1–S4, Tables S1 and S2, and Supplemental Experimental Procedures

Table S3. Related to Figures S1 and 4 Excel file containing a complete list of peptides identified in the Chl4-SZZ, Mcm21-SZZ, Scc2-SZZ, and Scc4-SZZ pull-downs shown in Figures S1E and S1F, together with no-tag controls, and a complete list of peptides identified in the Scc2-6His-3FLAG pull-downs from G1-arrested cells in the presence and absence of ectopic Scc1 production (Figure 4C).

Movie S1. Related to Figure 2. Wild-Type Cell Progressing from Late G1 through S Phase Live-cell imaging of Scc2-GFP and Mtw1-tdTomato of a wild-type cell progressing from late G1 through S phase, as shown in Figure 2E.

Movie S2. Related to Figure 2. Wild-Type Cell Progressing from Metaphase to Anaphase Live-cell imaging of Scc2-GFP and Mtw1-tdTomato of a wild-type cell progressing from metaphase to anaphase, as shown in Figure 2F.

Movie S3. Related to Figure 2. *chl4Δ* Cell Progressing from Late G1 through S Phase Live-cell imaging of Scc2-GFP and Mtw1-tdTomato of a *chl4Δ* cell progressing from late G1 through S phase, as shown in Figure 2G.

Movie S4. Related to Figure 2. *chl4Δ* Cell Progressing from Metaphase to Anaphase Live-cell imaging of Scc2-GFP and Mtw1-tdTomato of a *chl4Δ* cell progressing from metaphase to anaphase, as shown in Figure 2H.
