# Supplementary material for: Cohesin-Dependent Association of Scc2/4 with the Centromere Initiates Pericentromeric Cohesion Establishment
Source: Curr Biol. 2013 Apr 8;23(7):599–606. doi: 10.1016/j.cub.2013.02.022 (PMC3627958; doi:10.1016/j.cub.2013.02.022)
Supplement: Document S1. Figures S1–S4, Tables S1 and S2, and Supplemental Experimental Procedures [file mmc1.pdf]

Current Biology, Volume *23*

Supplemental Information

Cohesin-Dependent Association of Scc2/4 with the Centromere Initiates Pericentromeric Cohesion Establishment

Josefin Fernius, Olga O. Nerusheva, Stefan Galander, Flavia de Lima Alves, Juri Rappsilber, and Adele L. Marston

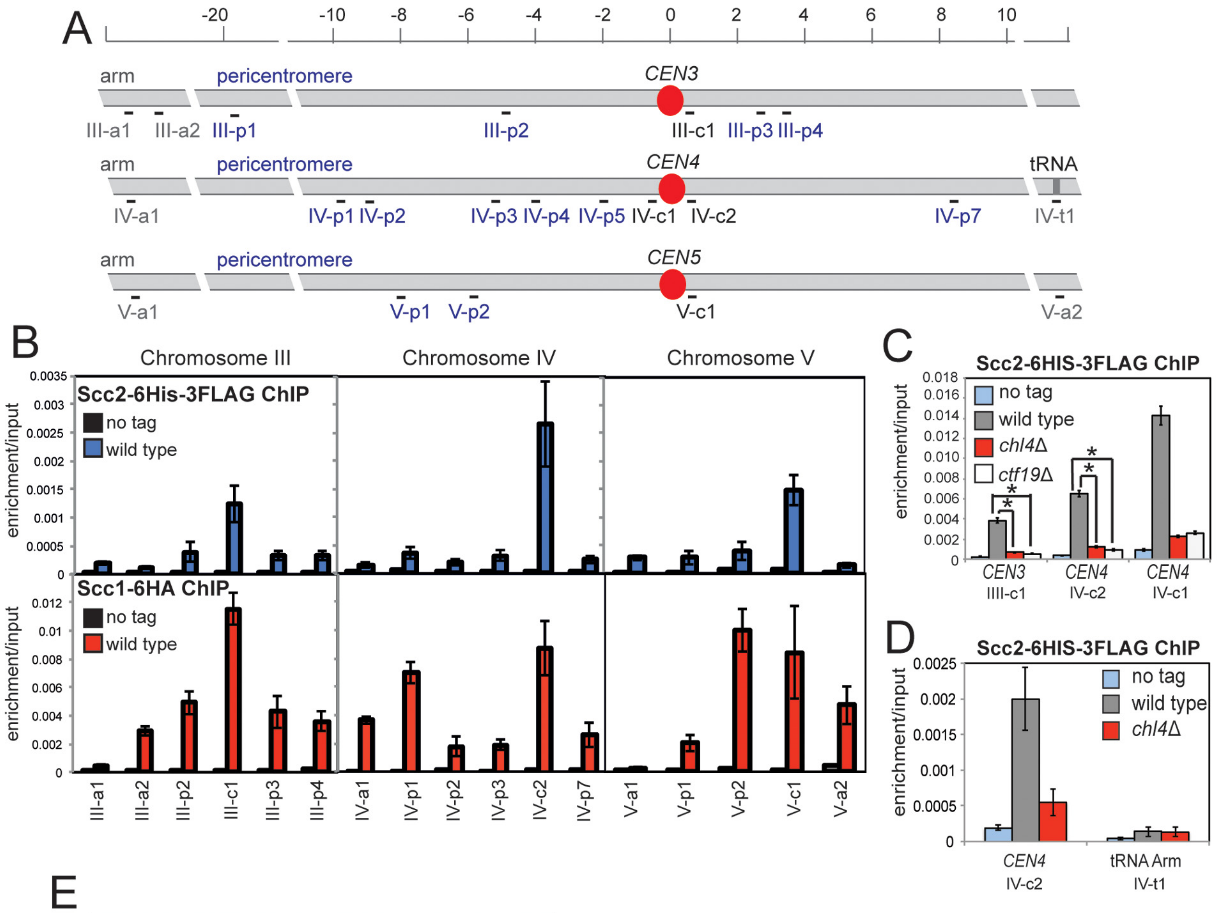

E

Subcomplex	Protein	Number of peptides Chl4-SZZ	SMC3E1155Q Mcm21-SZZ
Mif2	Mif2 (C)	6	4
Ctf19/CCAN	Mcm21 (O)	30	**43**
	Ctf19 (P)	37	49
	Okp1 (Q)	32	39
	Ame1 (U)	21	22
	Mcm16 (H)	10	11
	Ctf3 (I)	56	37
	Mcm22 (K)	23	17
	Cnn1 (T)	5	5
	Chl4 (N)	**52**	28
	Iml3 (L)	30	14
	Nkp1	18	19
	Nkp2	8	6
Ndc80	Ndc80/Tid3	31	22
	Nuf2	13	6
	Spc24	3	3
	Spc25	8	7
MIND	Mtw1	14	12
	Nsl1	5	10
	Nnf1	9	8
	Dsn1	19	18
Spc105	Spc105	9	12
	Ydr532p	5	4
Dam1	Dam1	4	0
	Duo1	5	1
	Spc34	5	4
	Spc19	2	1
	Ask1	3	0
CEN nucleosome	Cse4	5	4
	Hhf1	8	1
	Hta1/2	5	2
	Htb1/2	7	3
Cohesin loader	Scc2	0	2
	Scc4	0	0
Cohesin	Smc1	0	2
	Smc3	0	2
	Scc1/Mcd1	0	0
	Scc3/Irr1	0	0
	Pds5	0	0

F

subcomplex	Protein	number of peptides Scc2	Scc4
cohesin loader	Scc2	144	131
	Scc4	60	55
cohesin	Smc3	20	14
	Smc1	20	4
	Scc1/Mcd1	8	6
	Pds5	8	
	Scc3/Irr1	6	2

Figure S1, Related to Figure 1. The Ctf19 Complex Is Required for Scc2 Enrichment at the Centromere

(A) Schematic diagram showing the positions of primer sets.

(B) Centromeres are the strongest binding sites for Scc2. Analysis of Scc2-6His-3FLAG (upper graph) or Scc1-6HA (lower graph) association at the indicated sites by ChIP-qPCR. Strains AM1105 (*SCC1-6HA*) or AM6753 (*SCC2-6HIS-3FLAG*) and AM2508 (no tag control) all carrying *pMET-CDC20* were arrested in G1 using alpha factor and released into medium containing methionine (to deplete Cdc20) together with nocodazole and benomyl (to depolymerize microtubules). Cells were harvested 2 hours after release from G1. Anti-HA or anti-FLAG antibodies were used for ChIP and samples analyzed by qPCR with primers at the indicated sites on chromosomes III, IV and V.

(C and D) Analysis of Scc2 levels at centromeres in cells lacking Ctf19 components and arrested in metaphase of mitosis without microtubules. Strains AM1176 (no tag), AM6006 (*SCC2-6HIS-3FLAG*), AM7897 (*SCC2-6HIS-3FLAG chl4Δ*) and AM7899 (*SCC2-6HIS-3FLAG ctf19Δ*) were treated with nocodazole for 3 h and harvested for ChIP-qPCR. Mean values of three independent experiments are shown and error bars indicate standard deviation in B, C and D. * $p<0.05$ paired two-tailed t-test.

(E) Proteomic analysis of Ctf19 complex-associated proteins. The number of peptides of the indicated proteins identified by mass spectrometry after immunoprecipitation of Chl4-SZZ or Mcm21-SZZ from strains AM3279 (*CHL4-SZZ*) and AM9708 (*MCM21-SZZ SMC3-E1155Q-3HA*) as described in materials and methods.

(F) The cohesin loader associates with cohesin in cycling cells. Peptides of cohesin and its loader identified by mass spectrometry after purification of Scc2-SZZ(TAP) or Scc4-SZZ(TAP) from cycling cells of strains AM8219 and AM8317.

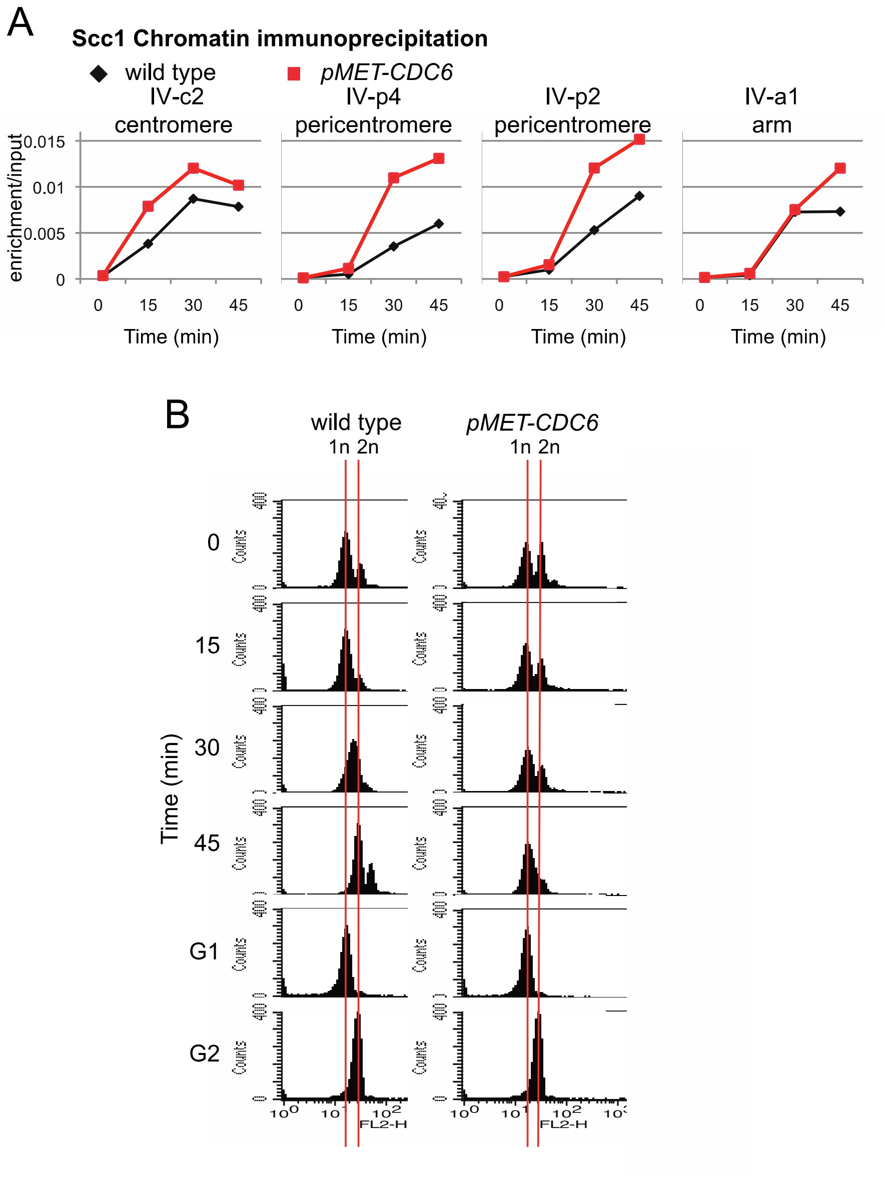

Figure S2, Related to Figure 2. Loading of Scc1 Does Not Require Replication
Analysis of Scc1 loading at centromeres in the absence of replicated chromosomes. Strains AM1145 (*SCC1-6HA*) and AM5632 (*SCC1-6HA, pMET3-CDC6*) were grown overnight in synthetic media lacking methionine to allow expression of *CDC6*. Cells were then arrested in G1 with alpha factor in media lacking methionine for 2.5 hours. The alpha factor was then washed out and the cells released into media lacking methionine. 20 minutes after release from G1, 8 mM methionine was added to the media to shut off expression of *CDC6*. 45 minutes after release from G1 when small buds started to appear, alpha factor was added to arrest them in G1 for 2 hours. The cells were again washed to allow release in media containing 8mM methionine to allow entry into the cell cycle in the absence of Cdc6. Scc1-6HA ChIP and FACS samples were taken at G1 and 15, 30 and 45 minutes following release from G1.

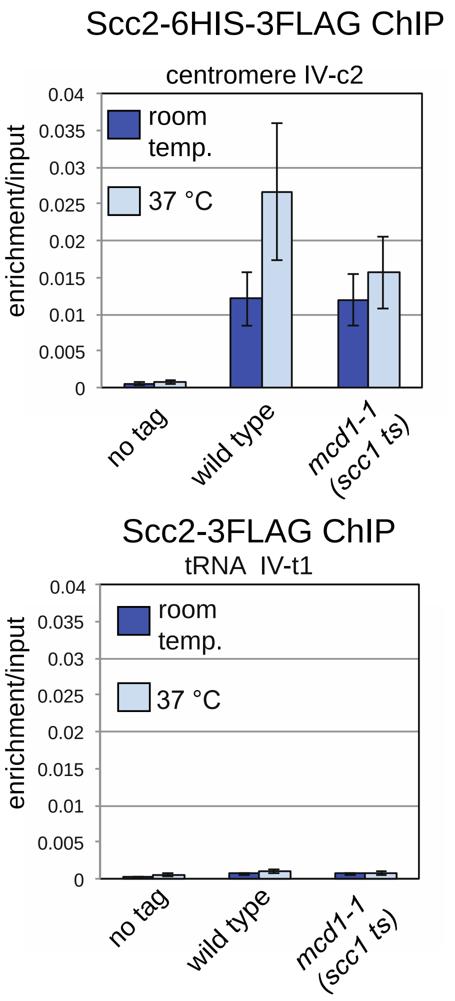

Figure S3, Related to Figure 3. Reduced Association of Scc2 with Centromeres at 37°C in *mcd1-1* Mutants Compared To Wild-Type Cells
Strains AM1176 (no tag), AM6006 (*SCC2-6HIS-3FLAG*) and AM9945 (*SCC2-6HIS-3FLAG mcd1-1*) were harvested after treatment with nocodazole and benomyl either at room temperature or 37 °C for 2h and Scc2 levels were analysed by ChIP-qPCR at the indicated sites.

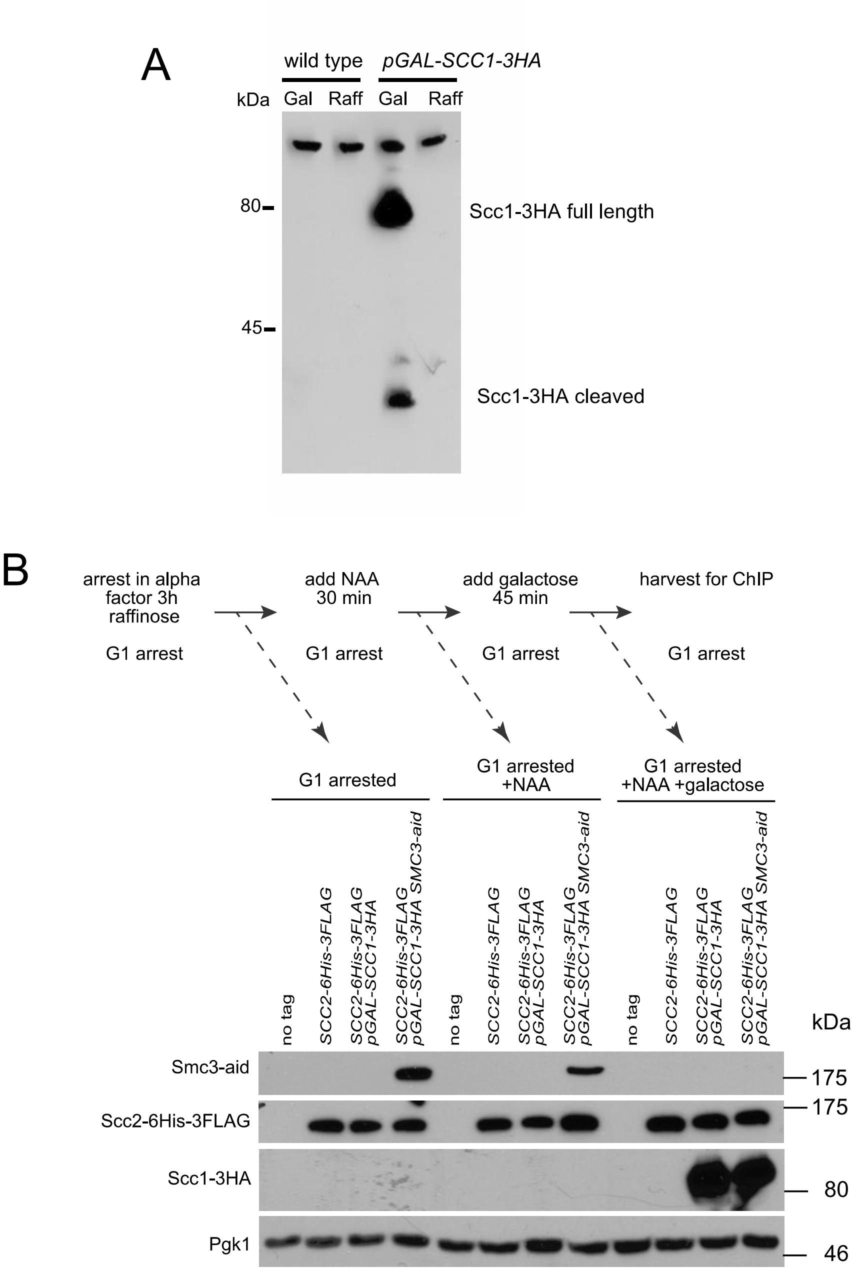

Figure S4, Related to Figure 4.
(A) Full length Scc1 production in G1 cells after ectopic expression of *SCC1-3HA*. Anti-HA immunoblot of a representative experiment carried out as described in Figure 4B is shown with the position of full length and cleaved Scc1 indicated.
(B) Scc1 and Scc2 are stable upon Smc3-aid degradation. Experimental set up and anti-FLAG, anti-HA, anti-aid and anti-Pgk1 western blot for the experiment shown in Figure 4D. Pgk1 is shown as a loading control.

Table S1. Yeast Strains

Strain Number	Relevant Genotype
AM1105	*MATa pMET-CDC20 SCC1-6HA*
AM1145	*MATa SCC1-6HA*
AM1176	*MATa* W303 wild-type
AM2508	*MATa pMET-CDC20*
AM3276	*MATa CHL4-6HA*
AM4643	*MATa pMET-CDC20 CEN4(2.4kbR)::tetOs tetR-GFP SPC42-tdTomato*
AM4644	*MATa pMET-CDC20 CEN4(2.4kbR)::tetOs tetR-GFP SPC42-tdTomato chl4Δ*
AM5632	*MATa SCC1-6HA pMET-CDC6*
AM6004	*MATa leu2::pGAL-SCC1-3HA::LEU2, chl4Δ*
AM6006	*MATa SCC2-6HIS-3FLAG*
AM6534	*MATa SCC2-SZZ (TAP)*
AM6753	*MATa pMET-CDC20 SCC2-6HIS-3xFLAG*
AM7897	*MATa SCC2-6HIS-3xFLAG, chl4Δ*
AM8184	*MATa leu2-3, trp1-1, ura3-52, prb1, prc1, pep4-4*
AM8219	*MATa leu2-3, trp1-1, ura3-52, prb1, prc1, pep4-4 SCC2-SZZ(TAP)*
AM8220	*MATa leu2-3, trp1-1, ura3-52, prb1, prc1, pep4-4 CHL4-SZZ(TAP)*
AM8317	*MATa leu2-3, trp1-1, ura3-52, prb1, prc1, pep4-4 SCC4-SZZ(TAP)*
AM8387	*MATa CEN4(500bpR)::lacOs(10kb)::LEU2*
AM8413	*MATa SCC2-6HIS-3FLAG SCC1-6HA*
AM8414	*MATa SCC2-6HIS-3FLAG, SCC1-6HA*
AM8415	*MATa SCC2-6HIS-3FLAG SCC1-6HA, chl4Δ*
AM9334	*MATa SCC2-6HIS-3FLAG, leu2::pGAL-SCC1-3HA::LEU2*
AM9335	*MATa SCC2-6HIS-3FLAG*

Strain Number	Relevant Genotype
AM9519	*MATa SCC2-6HIS-3FLAG pMET-SCC1myc18::TRP1*
AM9613	*MATa SCC2-6HIS-3FLAG leu2::pGAL-SCC1-3HA::LEU2 chl4Δ*
AM9708	*MATa leu2::SMC3-3HA(E1155Q)::LEU2 MCM21-SZZ(TAP)*
AM9945	*MATa SCC2-6HIS-3FLAG mcd1-1*
AM10285	*MATa CEN4(500bpR)::lacOs(10kb)::LEU2 SCC2-6HIS-3FLAG SCC1-6HA*
AM10287	*MATa CEN4(500bpR)::lacOs(10kb)::LEU2 SCC2-6HIS-3FLAG SCC1-6HA ura3::pGAL-SCC4-lacI::URA3*
AM10289	*MATa CEN4(500bpR)::lacOs(10kb)::LEU2 SCC2-6HIS-3FLAG SCC1-6HA chl4Δ*
AM10291	*MATa CEN4(500bpR)::lacOs(10kb)::LEU2 SCC2-6HIS-3FLAG SCC1-6HA chl4Δ ura3::pGAL-SCC4-lacI::URA3*
AM10570	*MATa pMET-CDC20 CEN4(500bpR)::lacOs(10kb)::LEU2 lacI-GFP*
AM10571	*MATa pMET-CDC20 CEN4(500bpR)::lacOs(10kb)::LEU2 lacI-GFP ura3::pGAL-SCC4-lacI::URA3*
AM10572	*MATa pMET-CDC20 CEN4(500bpR)::lacOs(10kb)::LEU2 lacI-GFP chl4Δ*
AM10573	*MATa pMET-CDC20 CEN4(500bpR)::lacOs(10kb)::LEU2 lacI-GFP chl4Δ ura3::pGAL-SCC4-lacI::URA3*
AM10713	*MATa/α SCC2-yeGFP/SCC2-yeGFP MTW1-tdTomato/MTW1-tdTomato*
AM10714	*MATa/α SCC2-yeGFP/SCC2-yeGFP MTW1-tdTomato/MTW1-tdTomato chl4Δ/chl4Δ*
AM10715	*MATa/α SCC2-yeGFP/SCC2-yeGFP MTW1-tdTomato/MTW1-tdTomato chl4Δ/chl4Δ leu2::pGAL-SCC1-3HA::LEU2/+*
AM10716	*MATa/α SCC2-yeGFP/SCC2-yeGFP MTW1-tdTomato/MTW1-*

Strain Number	Relevant Genotype
	tdTomato leu2::pGAL-SCC1-3HA::LEU2/+
AM11065	*MATa MATa SCC2-6HIS-3FLAG SCC1-6HA chl4Δ pGAL-SCC4-lacI*
AM11066	*MATa SCC2-6HIS-3FLAG SCC1-6HA pGAL-SCC4-lacI*
AM11092	*MATa SCC2-6HIS-3FLAG leu2::pGAL-SCC1-3HA::LEU2 SMC3-aid ura3::pADH1-OsTIR1-9Myc*
AM11162	*MATa pMET-CDC20 CEN4(2.4kbR)::tetOs tetR-GFP pGAL-SCC4-lacI*
AM11163	*MATa pMET-CDC20 CEN4(2.4kbR)::tetOs tetR-GFP chl4Δ pGAL-SCC4-lacI*
K13561	*MATa leu2::SMC3-3HA(E1155Q)::LEU2*
CB386	*MATa SCC2-6HIS-3FLAG*

Table S2. qPCR Primers

Primer set	AM Primer	Sequence
IV-a1	782	AGATGAAACTCAGGCTACCA
	783	TGCAACATCGTTAGTTCTTG
IV-p2	786	ATTGTTTAGAAACGGGAACA
	787	GTTCAACTCTCTGCATCTCC
IV-p4	788	GGTTGGGATCTAGGGATTAC
	789	TGATTGATTCACCTAGCCTT
IV-c1	792	ACACGAGCCAGAAATAGTAAC
	793	TGATTATAAGCATGTGACCTTT
IV-c2	794	CCGAGGCTTTCATAGCTTA
	795	ACCGGAAGGAAGAATAAGAA
IV-p6	798	TCCGAGAGAGATTACAATGG
	799	TGCCATTAGGATGAAACTCT
V-c1	945	TGAAGGTGAGCTTAAGACAG
	946	CAACCATGTTCGTAGCTAAA
V-a1	949	CTACGGTAAATCTGGGTAGG
	950	TCCACTATCAAGTCACCAGA
V-a2	975	TTCTATCGCGTTTAAGTGTG
	976	TAGTTGATCGTATGGCAGAG
V-p1	983	TAGCTAATGGAAATTATGCG
	984	TAGTGCACCAGATAAAGCAC
V-p2	985	TTCAATAATTGTGCCTTCTTC
	986	AGAACTCGCATGTGTCAAT
III-c1	1279	TGTTGATGGGTTTACAATTT
	1280	CTTTCAATGATTGCTCTAAATC
III-a2	1283	AGGCCAGTAGAAGAAGACTG
	1284	ATGGAATTTCACTTGGACAT
III-a1	1285	ATGGTACCTAGCTCGTGAAT
	1286	GGATTTGTCAACTTGGAACT
III-p2	1299	CCAATTTCTCAATTCACTCC
	1300	GCATTGCTTGTTATAGTGGA
III-p3	1301	AGAGAAGCAAGTTACGGTGT
	1302	AGCTCATATGTCTAGCAACG

Primer set	AM Primer	Sequence
III-p4	1303	CACCGTTTATTTCAATCTCA
	1304	TCCTTATGATTCGGAAGAAC
IV-p1	1319	ATGATTCAATGGATTTAGCC
	1320	GTCAGTCTTATGCTGTTCCC
IV-p3	1327	AGATCCCTGTAGACTGGATG
	1328	ACGCGTTTGAAGAACATT
IV-p5	1333	TGAAATCCTTCCTTTCTCAG
	1334	AATCGCATTATCAAACAGAA
IV-t1	1349	GACTTAATCCCAATATCGCT
	1350	CGACATATAATAGGCGAACA
IV-p7	1404	ATTTCGAATAAAGAGCATGG
	1405	CACACTTCACCAAGAACAAG
III-p1	3224	AACATACCACGTTGAACGGA
	3225	GCGATATCCCTTAGTGCCAT

Supplemental Experimental Procedures

Yeast Strains, Plasmids and Growth Conditions

Yeast strains used are shown in Table S1 and are all derivatives of w303 except protease-deficient strains AM8184, AM8219, AM8220, which are s288c derivatives. The *chl4Δ* and *ctf19Δ* strains were described in [1]. TAP (SZZ)-tagged strains were generated using a PCR-directed method [2]. The *pGAL-3HA-SCC1*, *SCC2-6HIS-3FLAG*, *SMC3-3HA(E1155Q)* constructs were described in [3, 4] and [5] respectively. The *pMET-SCC1* construct was described in [6] and the *SCC1-6HA* construct was described in [7]. The *SCC2-yeGFP*, *MTW1-tdTomato* and *SMC3-aid* strains were constructed using a PCR-based method [2, 8, 9].

To integrate *lacO*s adjacent to *CEN4*, a ~350 bp fragment immediately to the right of *CEN4* was cloned into pAFS59 [10] to generate AMp824, which was transformed after *HpaI* digestion. A vector (AMp916) carrying *lacI* under control of *pGAL* was constructed to generate plasmid AMp918 (*pGAL-SCC4-lacI*), which was integrated into the *URA3* locus. *tetOs* at *CEN4* have been described previously [11].

Growth conditions for additional experiments are given in the figure legends. Raffinose and galactose were used at 2%, methionine was used at 8 mM and 4 mM was re-added every hour. Benomyl and nocodazole were used at 30 μg/ml and 15 μg/ml, respectively, and nocodazole was re-added at 7.5 μg/ml. NAA (synthetic auxin derivative) was used at 0.5 mM.

Microscopy and Live-Cell Imaging

Scoring of LacI-GFP/lacO foci was performed in formaldehyde-fixed samples as described by [1].

For live-cell imaging, cells were cultured in synthetic complete medium, loaded onto the Onix Microfluidic Perfusion system (CellAsic) and visualized using a Deltavision Elite (Applied Precision) coupled to a Cascade 2 EMCCD camera with temperature control to 30 °C. Frames were grabbed every 7 min. Images were processed in ImagePro and figures prepared in Adobe Photoshop.

Western Blotting and FACS

Western blotting was performed as described in [6]. Anti-FLAG M2 antibodies (Sigma), anti-lacI (Millipore), anti-aid (Cosmo Bio) and anti-HA (Cambridge BioScience Ltd.) antibodies were used at a dilution of 1:1000. FACS was performed as described in [1].

Chromatin Immunoprecipation and qPCR

Chromatin immunoprecipitation using anti-FLAG M2 (SIGMA) or anti-HA (clone 12CA05) antibodies was performed as described by [1] (Figure S1B) or with the following modifications. Immunoprecipitated and 1/100 input chromatin was recovered by boiling (10 min) with a 10% slurry of Chelex-100 resin before adding Proteinase K (0.125 mg) and incubating at 55 °C for 30 min. Samples were boiled again, centrifuged and the supernatant extracted for qPCR analysis on a Roche Lightcycler using Express SYBR green reagent (Invitrogen). The sequences of primers used for qPCR are given in Table S2.

Purification of Protein Complexes

For purification of the Ctf19 complex (Chl4-SZZ (TAP) or Mcm21-SZZ (TAP)), Scc2-SZZ, Scc4-SZZ or Scc2-6HIS-3FLAG, 2 L of cycling culture in YEP+adenine + glucose was harvested. For purification of Scc2-SZZ (TAP) from G1-arrested cells, cells at OD_{600}=0.2 were arrested in G1 in 2 L YEP + adenine + raffinose (2%) by treatment with alpha factor (5 μg/ml) for 3 h. Galactose was then added (2%) and cells were harvested after 45 min. Upon harvesting in a cold centrifuge, cells were washed once with cold water, then incubated with the crosslinker dithiobis (succunimidyl propionate) (DSP; Proteochem) to a final concentration of 2 mM in 20 ml DSP reaction buffer (20 mM HEPES, pH7.4; 100 mM potassium acetate) for 30 min at room temp. Cells were then pelleted by centrifugation, washed once in cold 10 mM Tris pH7.5 then once in cold dH_2O before resuspending in cold dH_2O to 20 % of the pellet volume and drop frozen into liquid nitrogen. Cell pellets (5 g-25 g) were ground to powder under liquid nitrogen in a Retsch ball mill. Cell powder was resuspended in 2x lysis buffer (100 mM bis-Tris propane, pH7.0, 200 mM KCL, 10 mM EGTA, 10 mM EDTA, 20 % glycerol) containing protease and phosphatase inhibitors (5 μg/ml each chymostatin, leupeptin, antipain, aprotinin, pepstatin A, E64, 1x Roche EDTA free complete protease inhibitor cocktail, 5 μM sodium azide, 20 mM β-glycerolphosphate, 2 μM microcystin, 0.4 M sodium orthovanadate); 1 ml was added per 1 g of cell powder. After thawing, Triton X-100 was added to 1% and samples were sonicated at 39% amplitude for 5 s four times with 15 s resting on ice and lysate was obtained by centrifugation at 4000 rpm. To pulldown TAP (SZZ)- tagged proteins, rabbit IgG was precoupled to epoxy-conjugated dynabeads (Invitrogen), following the manufacturers instructions. 1 mg of IgG-coupled dynabeads were incubated with lysate obtained from 5 g of cell powder for 45 min at 4 °C. For purification of FLAG-tagged proteins, lysates from 5 g of cell powder were pre-incubated with 35μl mouse anti-FLAG M2 antibody (Sigma) for 30 min before addition of 75 μl of Protein G dynabeads (Invitrogen) for a further 30 min. Immunoprecipitates were washed 5 times with 1x lysis buffer containing inhibitors. Protein complexes were eluted from the beads by addition of 25 μl 1x LDS loading dye (Invitrogen) and incubation at 70 °C for 10 min. The supernatant was transferred to a new tube, β-mercaptoethanol was added to 5% and samples were incubated at 90 °C for 5 min before loading onto polyacrylamide gels.

Mass Spectrometry

A band of coomassie-stained gel was excised and the proteins where digested using Trypsin as described elsewhere [12]. In brief, proteins were reduced in 10 mM DTT for 30 min at 37°C, alkylated in 55 mM iodoacetamide for 20 min at room temperature in the dark, and digested overnight at 37°C with 12.5 ng/μL Trypsin (Proteomics Grade, Sigma). The digestion media was then acidified to 0.1% of TFA and spun onto StageTips as described in the literature [13].Peptides were eluted in 20 μL of 80% acetonitrile in 0.1% TFA and were concentrated to 4 μL (Concentrator 5301, Eppendorf AG). The peptides sample was then diluted to 5 μL by 0.1% TFA for LC-MS/MS analysis.

Analyses were performed in a Velos LTQ-Orbitrap mass spectrometer (Thermofisher Scientific) coupled on-line to an Waters Nano AQUITY UPLC (Waters) or in a Q-Exactive mass spectrometer ((Thermofisher Scientific) coupled on-line to an

Ultimate 3000 RSLCnano System (Thermo Fisher Scientific). Injections were performed in an analytical column with a self-assembled particle frit [14] and C18 material (ReproSil-Pur C18-AQ 3 μm; Dr. Maisch, GmbH) was packed into a spray emitter (75-μm ID, 8-μm opening, 300-mm length; New Objective) using an air-pressure pump (Proxeon Biosystems). Mobile phase A consisted of water and 0.1% formic acid; mobile phase B, consisted of acetonitrile and 0.1% formic acid. The gradient used was 100 min. The peptides were loaded onto the column at a flow rate of 0.6 μL/min and eluted at a flow rate of 0.3 μL/min according to the gradient: 1 to 5% buffer B for 1 min, then to 32% B for 82 min, then to 35% B for 7 min and to 85% B for 5 min (Velos LTQ-Orbitrap); or at a flow rate of 0.5 μL/min, followed by elution at a flow rate of 0.2 μL/min according to the gradient: 2% to 40% buffer B for 168 min, then to 95% B for 11 min (Q-Exactive).

For the Velos LTQ Orbitrap, FTMS spectra were recorded at 60,000 resolution and the twenty most intense peaks of the MS scan were selected in the ion trap for MS2, (normal scan, wideband activation, filling 5.0E5 ions for MS scan, 1.0E4 ions for MS2, maximum fill time 100 ms, dynamic exclusion for 60 s). For the Q-Exactive, FTMS spectra were recorded at Normalized Colision energy of 25, 70,000 Resolution, AGC 1e6 and max filling time of 20 ms. The 10 most intense peaks of MS scan were selected in the ion trap for MS2, (17,500 resolution, AGC 1e6, maximum fill time 60 ms, dynamic exclusion for 60s). Searches were conducted against a database containing *Saccharomyces cerevisiae* sequences (SGD – *Saccharomyces* Genome Database). The search parameters were: MS accuracy, 6 ppm; MS/MS accuracy, 0.6 Da; enzyme, trypsin; allowed number of missed cleavages, 2; fixed modification, carbamidometylation on Cysteine; variable modification, oxidation on Methionine.

Supplemental References

1. Fernius, J., and Marston, A. L. (2009). Establishment of cohesion at the pericentromere by the Ctf19 kinetochore subcomplex and the replication fork-associated factor, Csm3. PLoS Genet. *5*, e1000629.
2. Longtine, M. S., McKenzie, A. 3., Demarini, D. J., Shah, N. G., Wach, A., Brachat, A., Philippsen, P., and Pringle, J. R. (1998). Additional modules for versatile and economical PCR-based gene deletion and modification in Saccharomyces cerevisiae. Yeast *14*, 953–961.
3. Uhlmann, F., and Nasmyth, K. (1998). Cohesion between sister chromatids must be established during DNA replication. Curr. Biol. *8*, 1095–1101.
4. Strom, L., Karlsson, C., Lindroos, H. B., Wedahl, S., Katou, Y., Shirahige, K., and Sjogren, C. (2007). Postreplicative formation of cohesion is required for repair and induced by a single DNA break. Science *317*, 242–245.
5. Arumugam, P., Nishino, T., Haering, C. H., Gruber, S., and Nasmyth, K. (2006). Cohesin's ATPase activity is stimulated by the C-terminal Winged-Helix domain of its kleisin subunit. Curr. Biol. *16*, 1998–2008.
6. Clift, D., Bizzari, F., and Marston, A. L. (2009). Shugoshin prevents cohesin cleavage by PP2A(Cdc55)-dependent inhibition of separase. Genes Dev. *23*, 766–780.
7. Megee, P. C., Mistrot, C., Guacci, V., and Koshland, D. (1999). The centromeric sister chromatid cohesion site directs Mcd1p binding to adjacent sequences. Molecular cell *4*, 445–450.
8. Knop, M., Siegers, K., Pereira, G., Zachariae, W., Winsor, B., Nasmyth, K., and Schiebel, E. (1999). Epitope tagging of yeast genes using a PCR-based strategy: more tags and improved practical routines. Yeast *15*, 963–972.
9. Nishimura, K., Fukagawa, T., Takisawa, H., Kakimoto, T., and Kanemaki M. (2009). An auxin-based degron system for the rapid depletion of proteins in nonplant cells. Nat Methods. *6*, 917-922.
10. Straight, A. F., Belmont, A. S., Robinett, C. C., and Murray, A. W. (1996). GFP tagging of budding yeast chromosomes reveals that protein-protein interactions can mediate sister chromatid cohesion. Curr. Biol. *6*, 1599–1608.
11. He, X., Asthana, S., and Sorger, P. K. (2000). Transient sister chromatid separation and elastic deformation of chromosomes during mitosis in budding yeast. Cell *101*, 763–775.
12. Shevchenko, A., Wilm, M., Vorm, O., and Mann, M. (1996). Mass spectrometric sequencing of proteins silver-stained polyacrylamide gels. Anal. Chem. *68*, 850–858.
13. Rappsilber, J., Ishihama, Y., and Mann, M. (2003). Stop and go extraction tips for matrix-assisted laser desorption/ionization, nanoelectrospray, and LC/MS sample pretreatment in proteomics. Anal. Chem. *75*, 663–670.
14. Ishihama, Y., Rappsilber, J., Andersen, J. S., and Mann, M. (2002). Microcolumns with self-assembled particle frits for proteomics. J Chromatogr A *979*, 233–239.
