# Supplementary figures and images for: Cohesin-Dependent Association of Scc2/4 with the Centromere Initiates Pericentromeric Cohesion Establishment
Source: Curr Biol. 2013 Apr 8;23(7):599–606. doi: 10.1016/j.cub.2013.02.022 (PMC3627958; doi:10.1016/j.cub.2013.02.022)

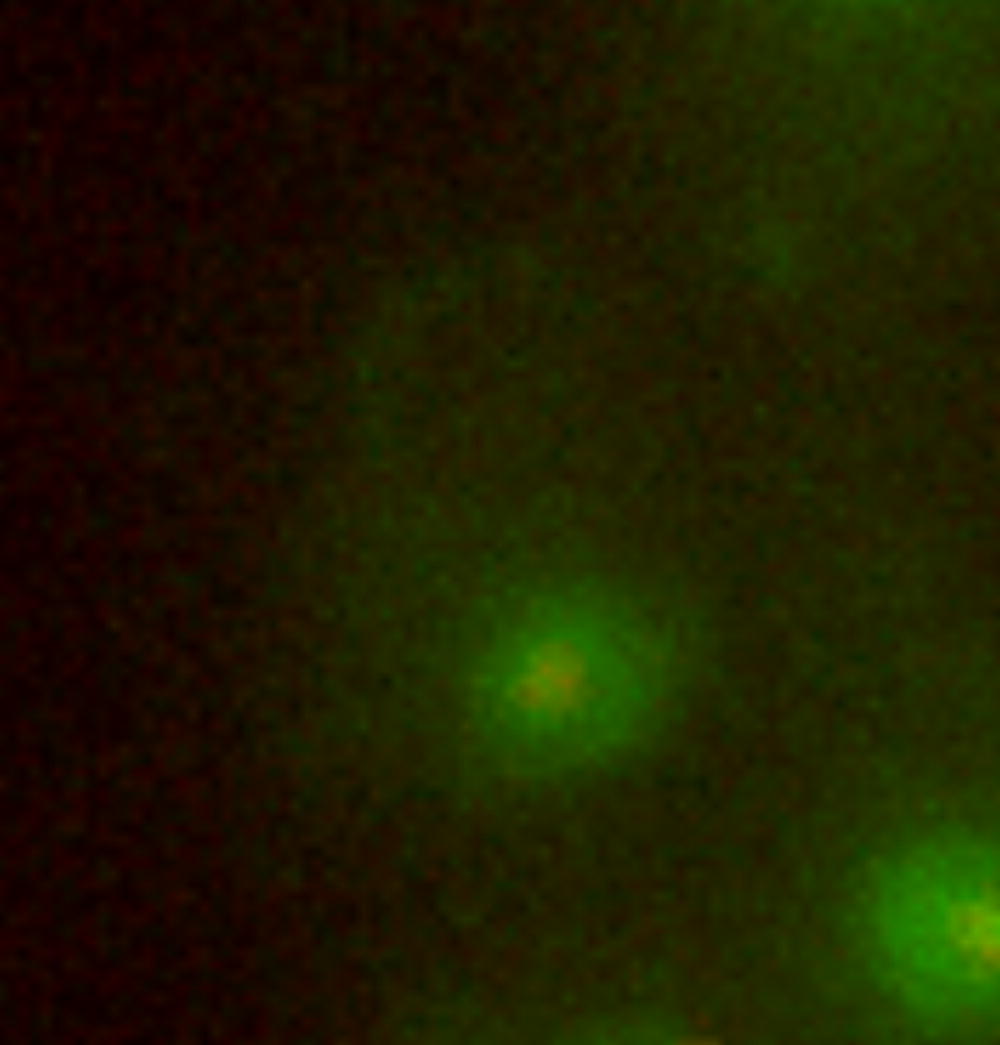

Supplement: Movie S1. Related to Figure 2. Wild-Type Cell Progressing from Late G1 through S Phase — Live-cell imaging of Scc2-GFP and Mtw1-tdTomato of a wild-type cell progressing from late G1 through S phase, as shown in Figure 2E. [file mmc3.jpg]

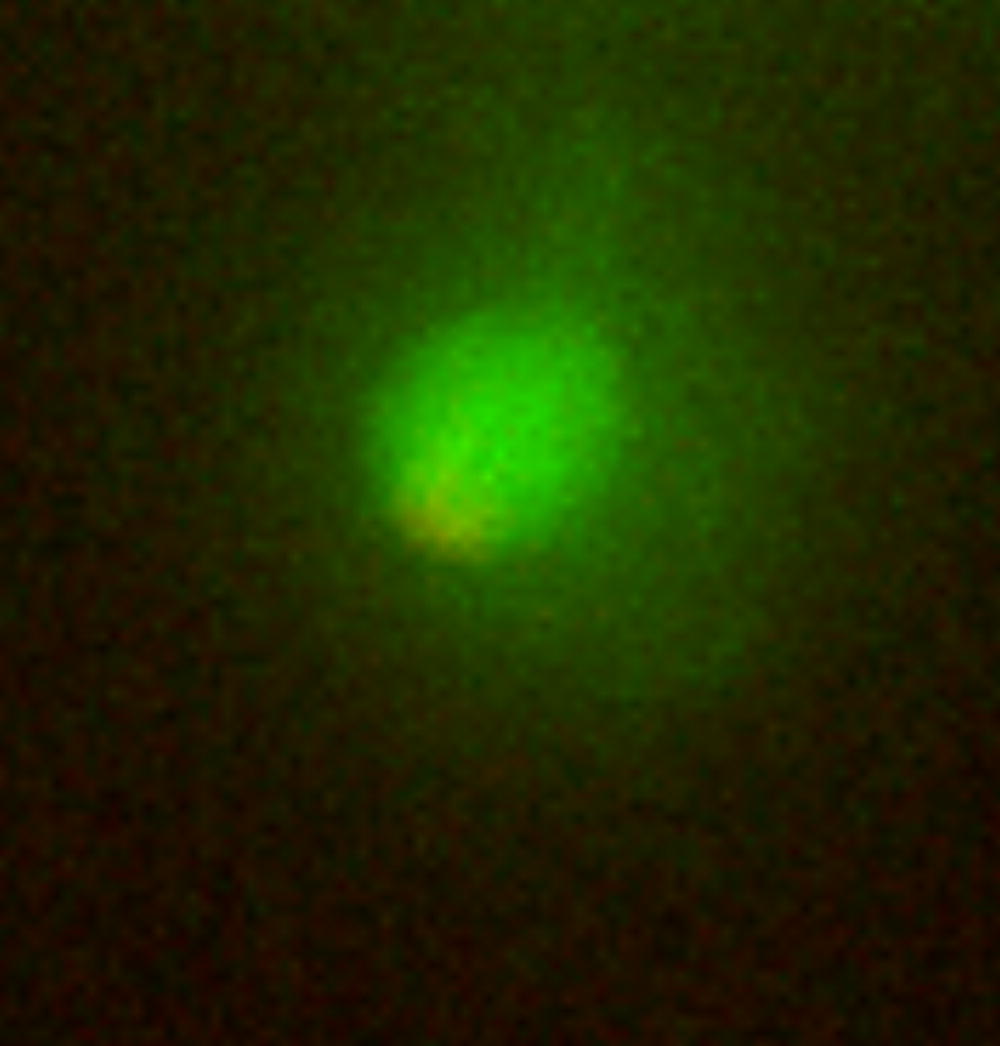

Supplement: Movie S3. Related to Figure 2. chl4Δ Cell Progressing from Late G1 through S Phase — Live-cell imaging of Scc2-GFP and Mtw1-tdTomato of a chl4Δ cell progressing from late G1 through S phase, as shown in Figure 2G. [file mmc5.jpg]

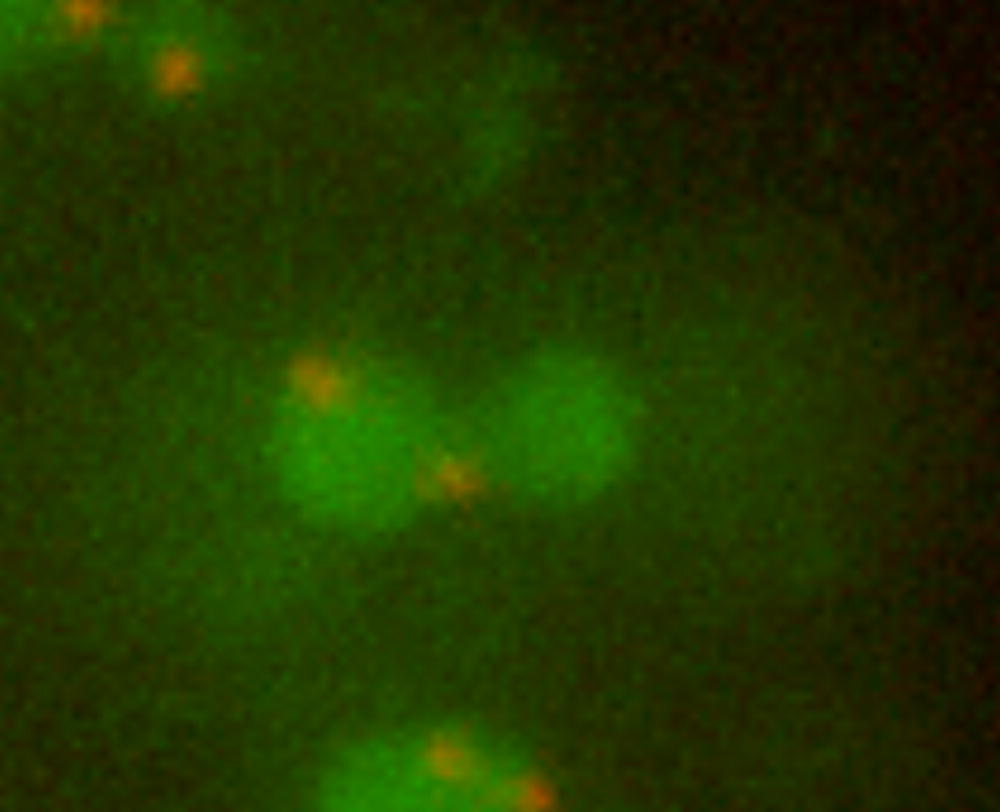

Supplement: Movie S4. Related to Figure 2. chl4Δ Cell Progressing from Metaphase to Anaphase — Live-cell imaging of Scc2-GFP and Mtw1-tdTomato of a chl4Δ cell progressing from metaphase to anaphase, as shown in Figure 2H. [file mmc6.jpg]
